# Supplementary material for: Transcriptomic Profiling of Shoot Apical Meristem Aberrations in the Multi-Main-Stem Mutant (ms) of Brassica napus L
Source: Genes (Basel). 2023 Jul 3;14(7):1396. doi: 10.3390/genes14071396 (PMC10378962; doi:10.3390/genes14071396)
Supplement: Supplementary file 1 [file genes-14-01396-s001.zip › supplemental figures 1-6.pdf]

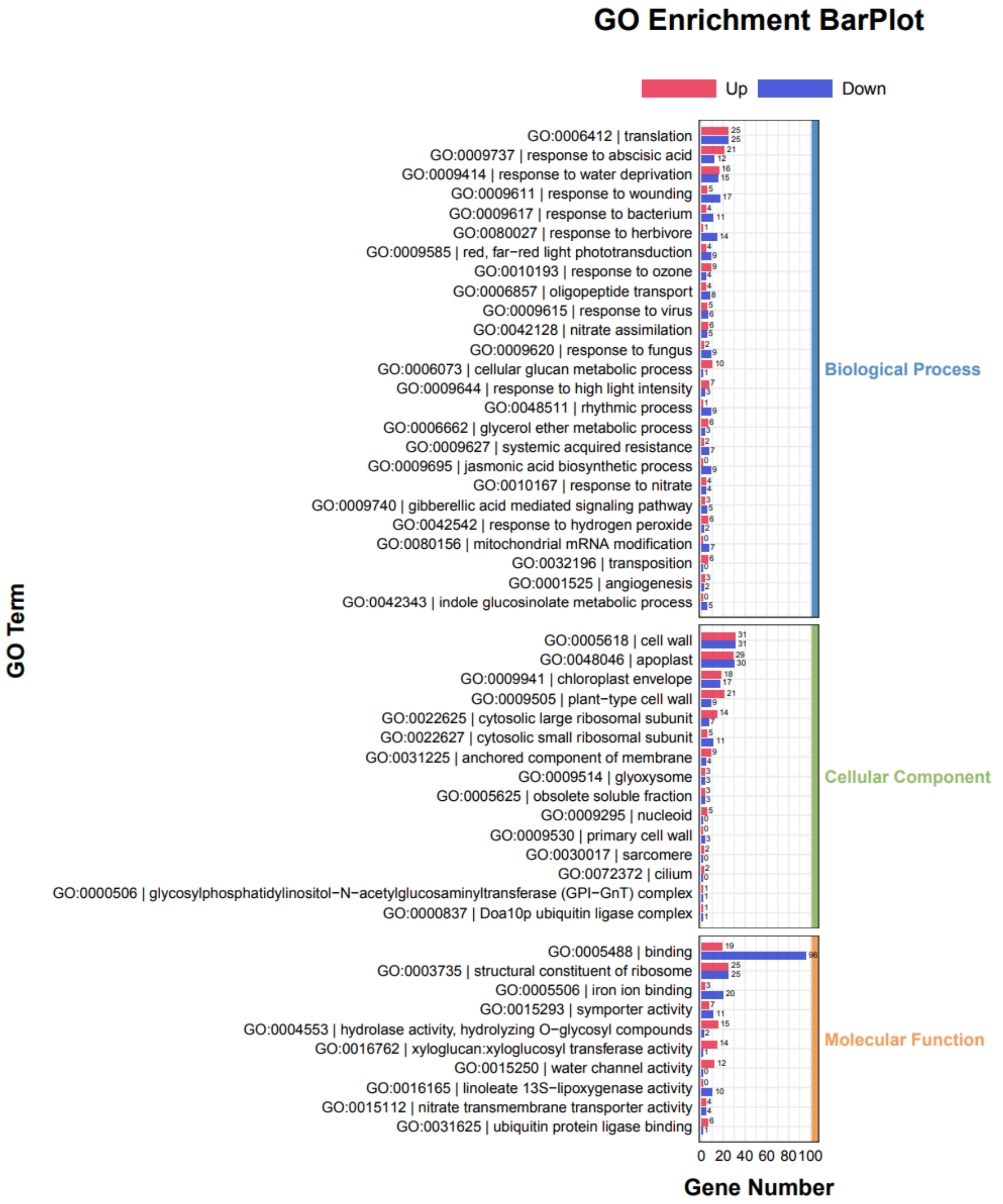

Figure S1. The GO enrichment analysis of DEGs between the *ms* mutant and WT at 15 days after germination (DAG).

All significantly enriched GO terms (*P* value < 0.05) based on biological process, cellular component, and molecular function enriched from the DEGs between the *ms* mutant and WT at 15 DAG. Numbers of up- and down- regulated DEGs of enriched GO terms are shown. Complete data can be found in Table S3.

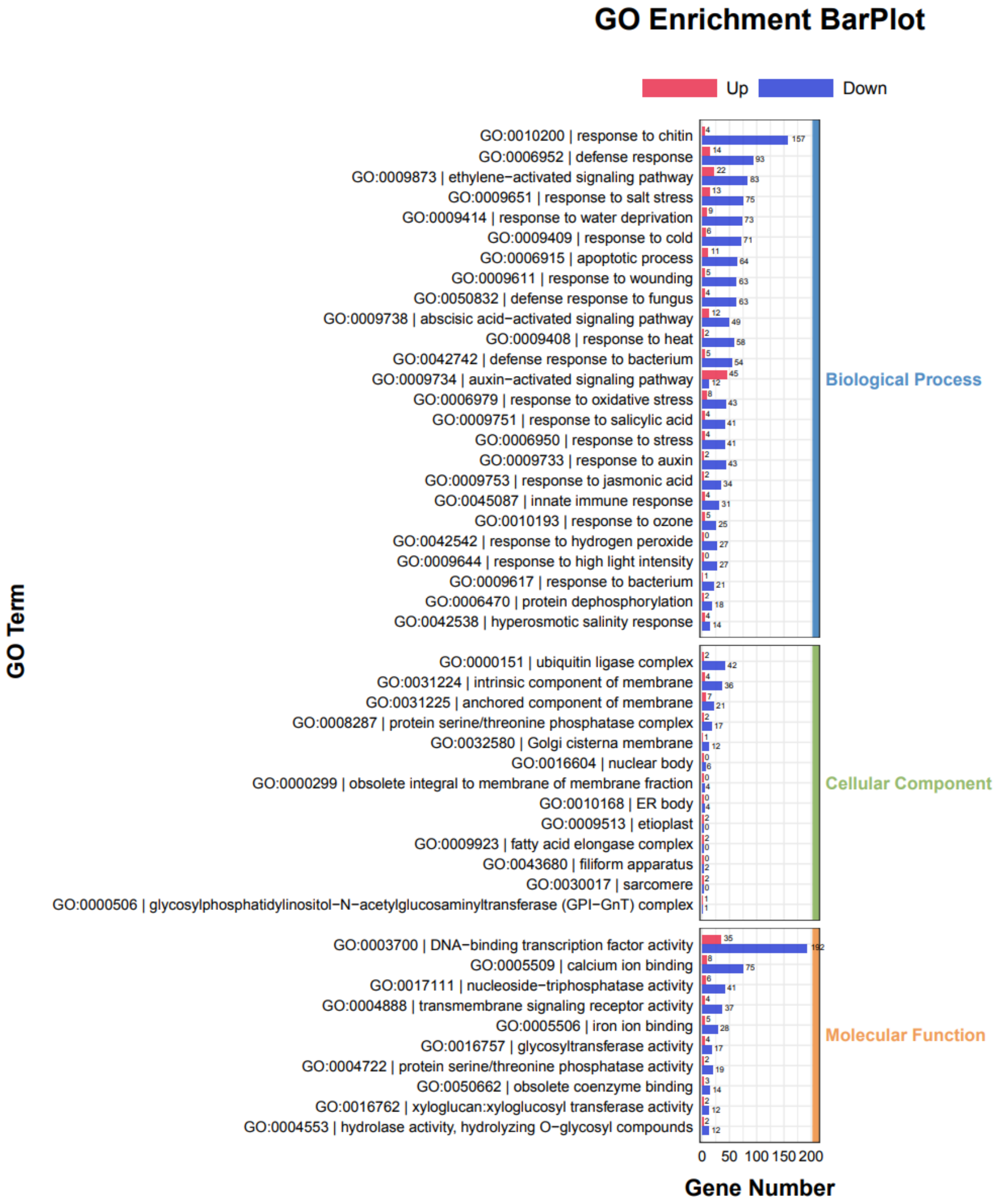

Figure S2. The GO enrichment analysis of DEGs between the *ms* mutant and WT at 20 days after germination (DAG).

All significantly enriched GO terms (P value < 0.05) based on biological process, cellular component, and molecular function enriched from the DEGs between the *ms* mutant and WT at 20 DAG. Numbers of up- and down- regulated DEGs of enriched GO terms are shown. Complete data can be found in Table S3.

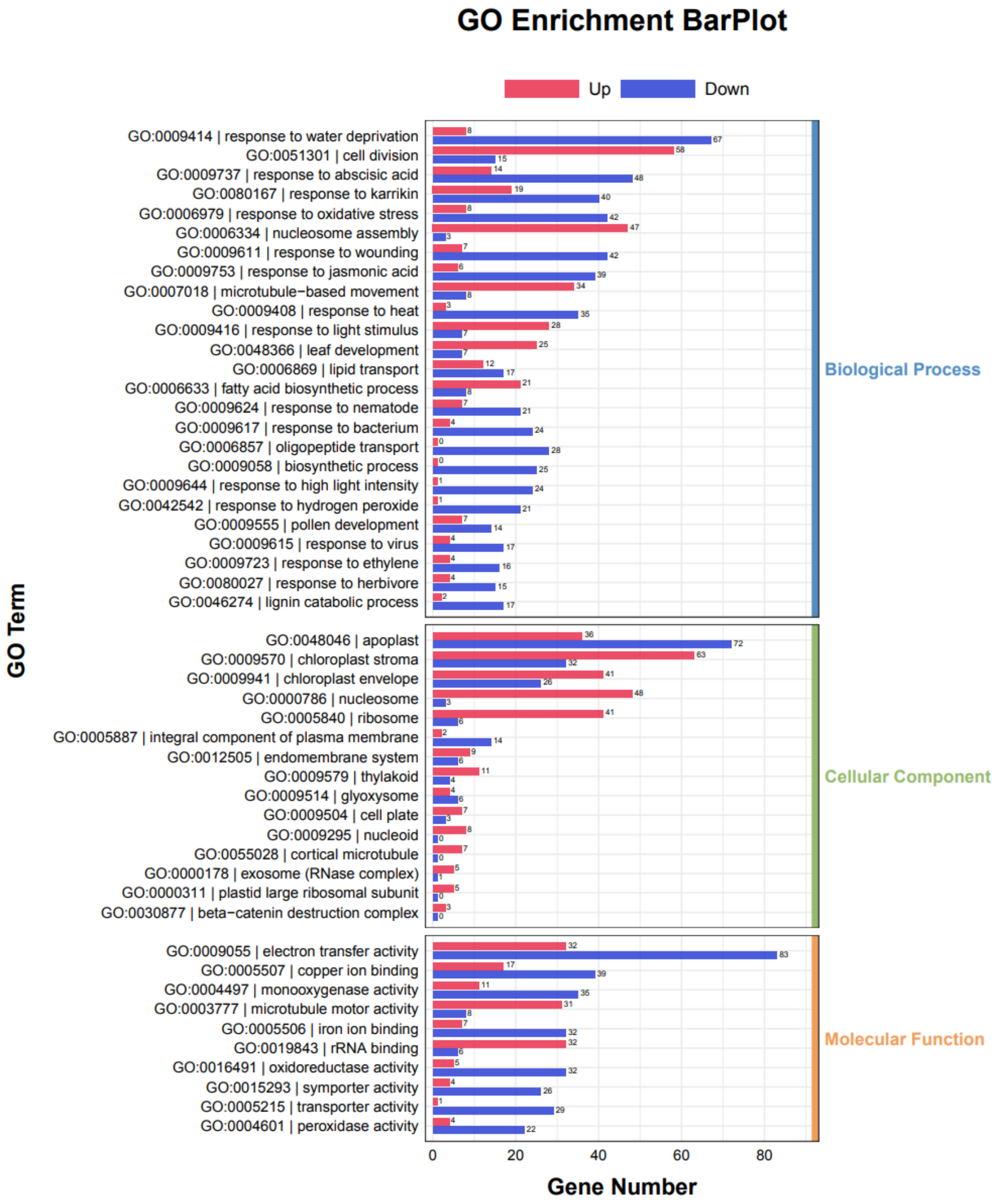

Figure S3. The GO enrichment analysis of DEGs between the *ms* mutant and WT at 25 days after germination (DAG).

All significantly enriched GO terms (*P* value < 0.05) based on biological process, cellular component, and molecular function enriched from the DEGs between the *ms* mutant and WT at 25 DAG. Numbers of up- and down-regulated DEGs of enriched GO terms are shown. Complete data can be found in Table S3.

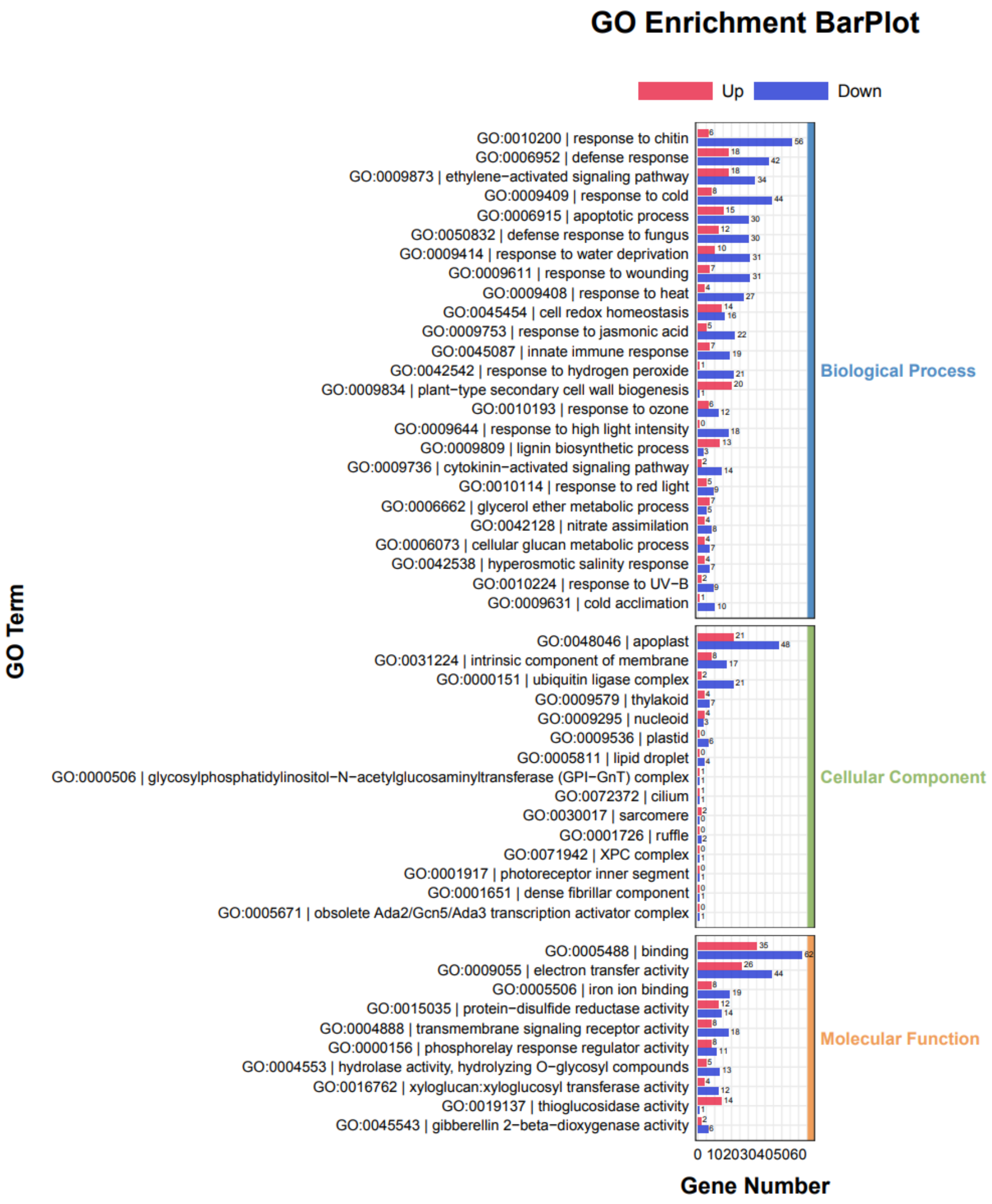

Figure S4. The GO enrichment analysis of DEGs between the *ms* mutant and WT at 30 days after germination (DAG).

All significantly enriched GO terms (P value < 0.05) based on biological process, cellular component, and molecular function enriched from the DEGs between the *ms* mutant and WT at 30 DAG. Numbers of up- and down-regulated DEGs of enriched GO terms are shown. Complete data can be found in Table S3.

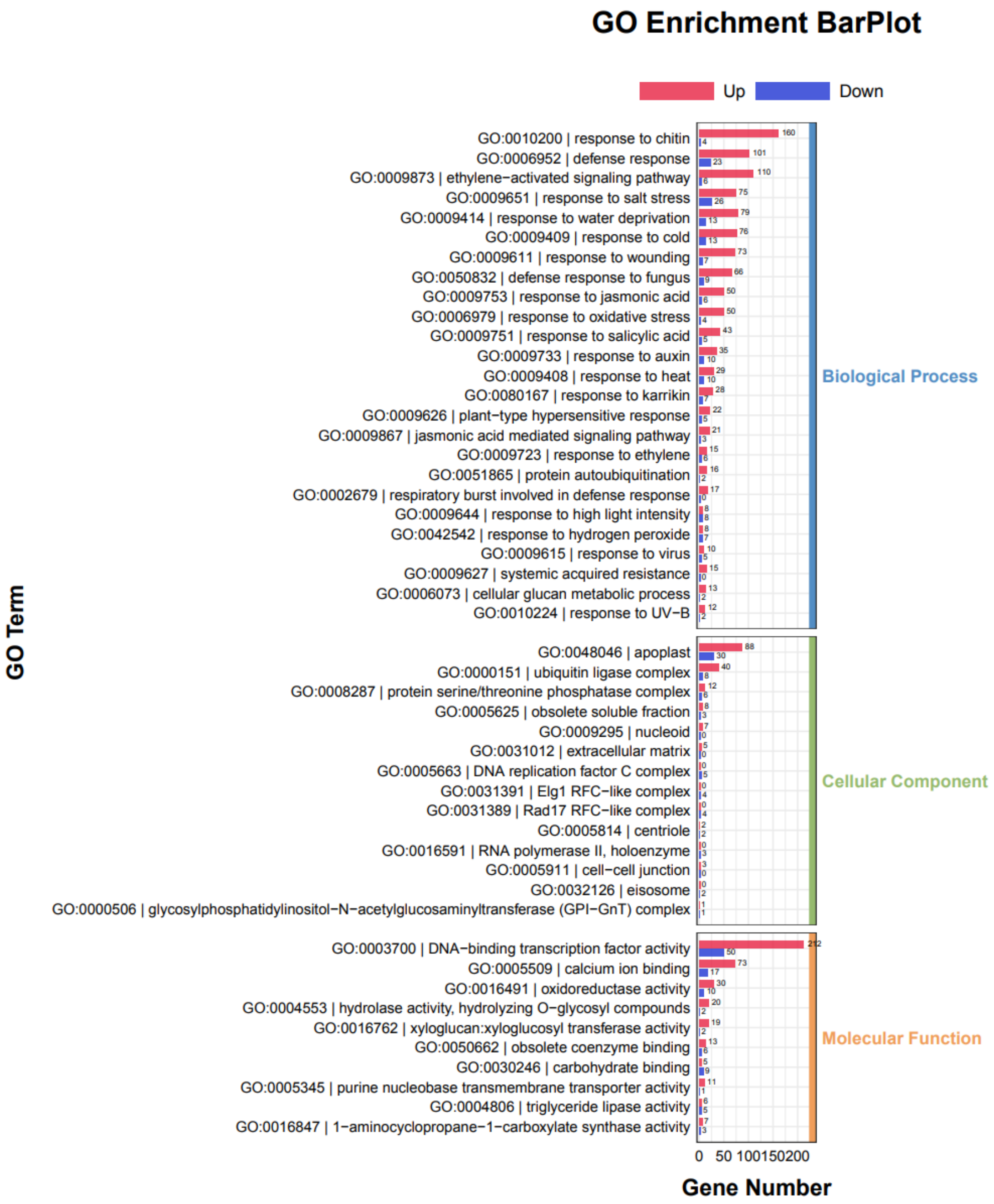

Figure S5. The GO enrichment analysis of DEGs between *ms* mutant and WT at 35 days after germination (DAG).

All significantly enriched GO terms (P value < 0.05) based on biological process, cellular component, and molecular function enriched from the DEGs between the *ms* mutant and WT at 35 DAG. Numbers of up- and down-regulated DEGs of enriched GO terms are shown. Complete data can be found in Table S3.

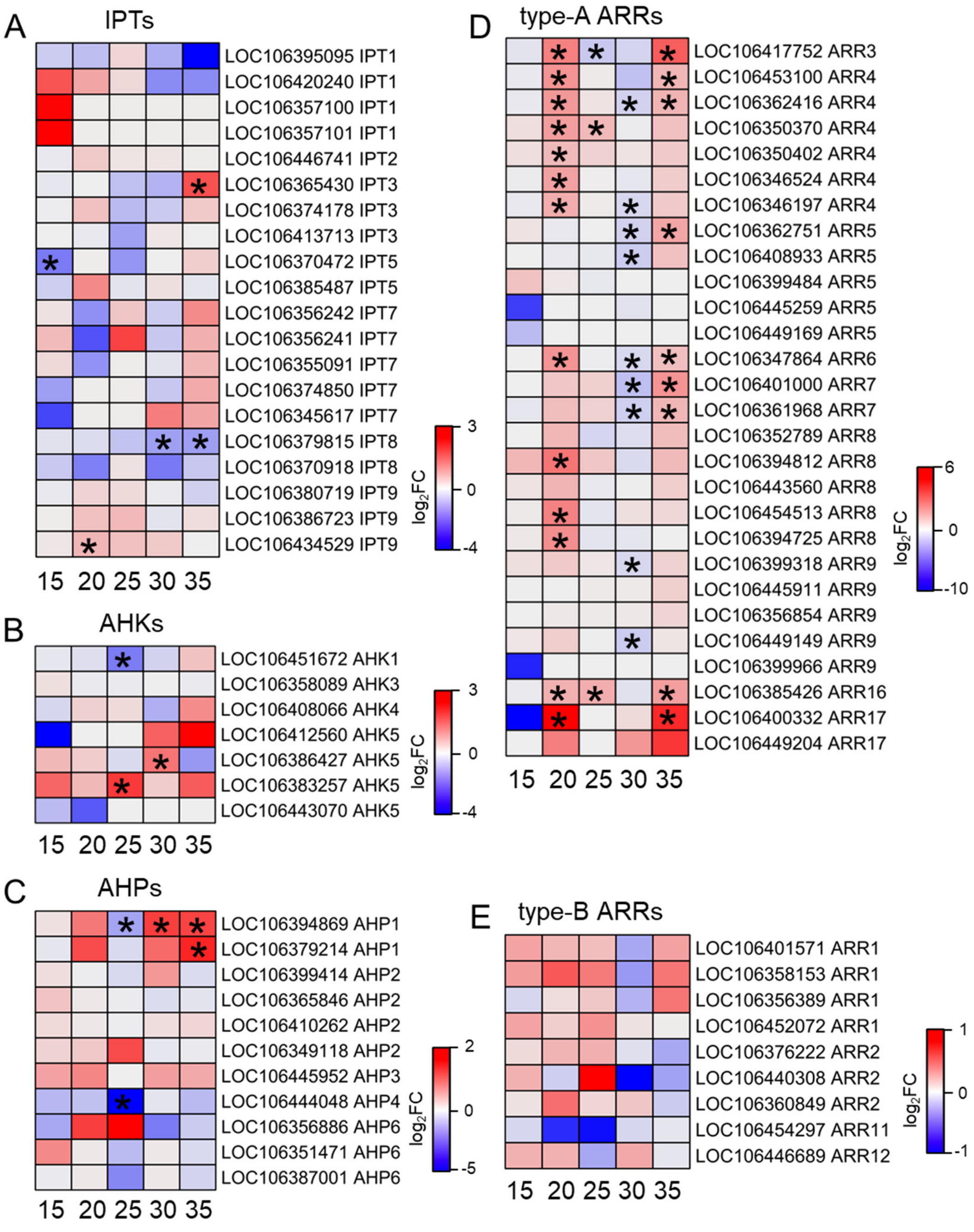

Figure S6. Relative transcript levels of genes involved in the CK signaling pathway.

(A) Heatmaps indicating the relative transcript levels ($\log_2$FC) of *IPTs*, (B) *AHKs*, (C) *AHPs*, (D) *type-A ARRs* and (E) *type-B ARRs* in the SAMs of the *ms* mutant compared with the WT at 15, 20, 25, 30, and 35 DAG, respectively. * highlights genes that were differentially expressed between the *ms* mutant and WT (p value < 0.05; $|\log_2(\text{fold change})| \geq 1$). Gene names and transcript levels are listed in Table S6.
